# Intratumoral NKp46^+^ natural killer cells are spatially distanced from T and MHC-I^+^ cells with prognostic implications in soft tissue sarcoma

**DOI:** 10.3389/fimmu.2023.1230534

**Published:** 2023-07-21

**Authors:** Sylvia M. Cruz, Cyrus J. Sholevar, Sean J. Judge, Morgan A. Darrow, Khurshid R. Iranpur, Lauren E. Farley, Marshall Lammers, Aryana M. Razmara, Cordelia Dunai, Alicia A. Gingrich, Julia Persky, Hidetoshi Mori, Steven W. Thorpe, Arta M. Monjazeb, William J. Murphy, Robert J. Canter

**Affiliations:** ^1^ Division of Surgical Oncology, Department of Surgery, University of California, Davis, Sacramento, CA, United States; ^2^ Department of Surgery, Memorial Sloan Kettering Cancer Center, New York, NY, United States; ^3^ Pathology and Laboratory Medicine, University of California, Davis, Sacramento, CA, United States; ^4^ Department of Dermatology, University of California, Davis, Sacramento, CA, United States; ^5^ Department of Surgical Oncology, MD Anderson Cancer Center, Houston, TX, United States; ^6^ Center for Immunology and Infectious Diseases, University of California, Davis, Sacramento, CA, United States; ^7^ Orthopedic Surgery, University of California, Davis, Sacramento, CA, United States; ^8^ Radiation Oncology, University of California, Davis, Sacramento, CA, United States

**Keywords:** NKp46, soft tissue sarcomas, tumor microenvironment, MHC-I, spatial localization, natural killer cell, CD56^dim^, CD56^bright^

## Abstract

**Introduction:**

Soft tissue sarcomas (STS) are rare, heterogenous malignancies with an unmet need for novel immunotherapies. Tumor infiltrating lymphocytes (TILs) have been linked with favorable outcomes in STS patients, though the contribution of natural killer (NK) cells and spatial relationships of TILs with MHC-I expressing cells lacks detailed characterization.

**Experimental design:**

Using archived and prospectively collected specimens, we evaluated intratumoral NK cells by immunohistochemistry (IHC), flow cytometry, and immunofluorescence (IF). We assessed spatial localization of NK and T cells by multiplex IF, analyzing the effects of MHC-I expression status on NK and T cell clustering.

**Results:**

Both intratumoral NKp46 and CD56^dim^ expression were associated with significantly improved overall survival (P=0.05), while higher infiltrates of CD56bright NK cells predicted a worse prognosis (P=0.05). The presence of intratumoral NK cells was inversely proportional to CD3^+^ T cells. Spatial analyses showed NK cells preferentially clustering close to other NK cells with sparse CD3^+^ T and CD8^+^ T cells in range (P<0.0001). Additionally, CD3^+^ T and CD8^+^ T cells showed significantly greater co-localization with MHC-I^+^ cells, compared to NK cells (P<0.0001). After neoadjuvant radiotherapy, there was greater CD8 clustering, while after neoadjuvant chemotherapy, there was overall lower TIL clustering.

**Conclusion:**

Intratumoral NK cells are prognostic in STS and localize closer to MHC-I- cells than T cells. Although both NK and T cells are associated with improved survival in STS, their differential distribution in the TME based on MHC-I expression status may serve as a biomarker for improved immunotherapy treatment selection.

## Introduction

Soft tissue sarcomas (STS) are a diverse family of mesenchymal tumors with the potential for metastatic dissemination and aggressive tumor biology (1). When metastases occur, treatment options are limited and survival is poor, underscoring the need for novel systemic therapies (2). Despite significant advances in immunotherapies in recent years for many solid tumors, STS have shown overall poor response rates to standard immune checkpoint immunotherapies (ICI), such as PD-1, PD-L1, and CTLA-4 blockade, in part due to their non-immunogenic, “cold” nature, with generally sparse immune infiltration and low PD-L1 expression (3, 4).

The majority of the currently available immunotherapies such as ICI and CAR-T cells act via T-cell-dependent pathways with the end result to supply or reinvigorate the antitumor properties of T-cells (5). In tumors such as STS, limited T cell infiltration is therefore viewed as a mechanism of resistance to ICI. The cornerstone of T cell activation occurs via the interaction of somatically rearranged T cell receptors (TCR) with foreign antigens presented by major histocompatibility (MHC) molecules (6). Antigen presentation, particularly via MHC-I to cytotoxic CD8^+^ T cells, is therefore a critical step in both priming and sensitizing T cells to recognize target cells such as tumors harboring tumor-associated or tumor-specific antigens. Among various immune evasion mechanisms, STS along with other cancers such as melanoma, breast, colorectal, and cervical cancers, have the propensity to downregulate tumor specific MHC-I in order to facilitate immune escape, contributing to both innate and acquired resistance to T-cell immunotherapies (7, 8). Natural killer (NK) cells, in contrast, are a subset of innate lymphocytes that can respond rapidly to malignantly-transformed and virally-infected cells without prior sensitization and can recognize tumor cells by reduced expression of MHC-I, a ligand that is inhibitory to NK cells in contrast to CD8^+^ T cells where it is essential for antigen presentation (9, 10). This feature of NK cells makes them a theoretically attractive modality of immunotherapy, especially in T-cell resistant tumors where MHC-I downregulation is present. While these principles appear self-evident, studies to date have not assessed these relationships in detail, especially with respect to co-localization of NK and T cells close to or separate from MHC-I expressing cells and each other in the TME.

Additionally, in contrast to T cells, NK cells show greater plasticity and diversity in their mechanisms of activation and inhibition. NK cells are regulated by a network of activating and inhibitory signals that are non-rearranged and germline encoded. Key NK receptors include killer immunoglobulin-like receptors, the NKG2 family of C-type lectin-like receptors, CD16, and natural cytotoxicity receptors (NCR), including NKp46, NKp44, and NKp30 (11, 12). NK cells provide rapid responses to target cells, as they have the ability to recognize and kill stressed cells in the absence of antigen presentation (12). NK cells have an extensive repertoire of receptors which regulate activation and inhibition which are heterogeneously expressed depending on environmental signals such as the tissue of residence and the inflammatory milieu. Although the tumor microenvironment (TME) is known to be hypoxic and nutrient poor, which can lead to downregulation of NKp46 and impaired effector functions (13, 14), it remains under debate whether NK cells in the TME are activated and tumor-reactive or are dysfunctional/exhausted because of the hostile environment of the TME (15).

However, since NK cells are governed by a balance of activating and inhibiting signals in the TME, it is likely that the NK cell populations in STS as well as other tumors are heterogenous, consisting of various phenotypes, that may respond differently to distinct tumor targets. NK cell education is classically thought to occur during NK cell development, however environmental components have been shown to alter licensing of mature NK cells (16), further highlighting the complexity and knowledge gap of NK subsets and their interaction with the hostile TME. Some evidence suggests that NKp46 is constitutively expressed on all NK cells (17), while others have demonstrated a population of NKp46^-^ NK cells (18), bringing to question how those two populations may be affected differently in the TME. CD3^-^CD56+ is a widely accepted phenotypic definition of human NK cells, and these NK cells can be further divided into CD56^bright^ and CD56^dim^ subsets, with CD56^bright^ primarily secreting cytokines and CD56^dim^ being predominantly cytotoxic (19, 20). While different NK cell phenotypes have been characterized to an extent based on known NK cells markers, evaluation of the clinical implications of each of these phenotypes has not been performed in detail. Closer examinations of these relationships may shed light on the respective roles of NK subsets in the TME and their complex interactions.

Herein, we aimed to determine the extent of intratumoral NK cell infiltration and assess the impact of different NK cell subsets on clinical outcomes, hypothesizing that intratumoral NK cells represent a heterogeneous population of NK cells in the TME with a spectrum of phenotypic and functional characteristics. Given the complex relationship and differential regulation T and NK cells by MHC-I, we sought to characterize the spatial localization of these immune constituents, hypothesizing that NK and T cells exhibit unique spatial localizations based on MHC-I expression and proximity to other immune effector cells.

## Methods

### Patient cohort and clinicopathologic data

We identified patients with STS who underwent surgical resection at the University of California, Davis, Medical Center between 2008-2022. Retrospective analysis of archived tumor specimens was approved per IRB Protocol #484670-4. Clinicopathologic data were collected on 130 patients, including age at diagnosis, sex, tumor histology subtype, tumor size, tumor site, tumor grade, neoadjuvant therapy, and patient disease and vital status. The primary endpoints were the occurrence of metastasis from the time of surgery, notated as metastasis-free survival (MFS), and the occurrence of patient death from the time of diagnosis, notated as overall survival (OS).

### Immunohistochemistry

Tissue microarrays (TMA) were constructed from formalin-fixed paraffin-embedded (FFPE) archived tumor tissue of 100 patients as described previously (21, 22). Immunohistochemical (IHC) staining was performed using the following antibodies: rabbit anti-human CD3 (Abcam Cat# ab16669, RRID : AB_443425), mouse anti-human CD8 (Agilent Cat# M7103, RRID : AB_2075537), mouse anti-human CD56 (Agilent Cat# M730429-2, RRID : AB_2750583), mouse anti-human HLA Class I ABC (MHC-I) (Abcam Cat# ab70328, RRID : AB_1269092), and rabbit anti-human NCR1 (NKp46) (Abcam, Cat# ab224703). Tumor infiltrating lymphocyte (TIL) and IHC expression scores were determined by a STS pathologist (MAD) in a blinded fashion, using techniques described previously (21). TIL scores were calculated on H&E-stained slides as follows: 3 (>20 TIL/high-power field (hpf)), 2 (11–20 TIL/hpf), 1 (1–10 TIL/hpf), 0 (<1 TIL/hpf). IHC expression scores for CD3, CD8, CD56, MHC-I, and NKp46 was calculated as follows to derive an H-score: [1x (% 1+cells)+2x(% 2+cells)+3x(% 3+cells)] where 1+, 2+, and 3+ represent weak, moderate, and strong stain intensity, respectively. TIL and IHC expression scores were averaged when more than one consecutive section was present for scoring (median 3 replicate cores per patient sample).

### Flow cytometry

Human tumor and blood samples (N=46) were collected prospectively from STS patients undergoing surgery under IRB approval at the University of California, Davis (protocol #939793-5). Samples were processed into single cell suspensions using lymphocyte separation for blood and mechanical digestion for tumors as described previously (21–23). Samples were stained using the following fluorochrome-conjugated monoclonal antibodies: CD3-FITC (BioLegend Cat# 300305, RRID : AB_314041), CD8-BV785 (BioLegend Cat# 301046, RRID : AB_2563264), CD45-BV510 (BioLegend Cat# 304035, RRID : AB_2561383), NCAM(CD56)-PE (BioLegend Cat# 318306, RRID : AB_604101), and NKp46(CD335)-BV605 (BioLegend Cat# 331926, RRID : AB_2563855). Live/dead staining was performed using Fixable Viability Dye 780 (eBioscience #65-0865-14). Samples were acquired using a flow cytometer (Fortessa LSR BD Biosciences, USA), and data were analyzed using FlowJo Software (Beckton Dickinson, San Jose, California, USA, RRID : SCR_008520).

### Multiplex immunofluorescence and imaging analysis

Multiplex immunofluorescence (mIF) was performed on 4 microns TMA tissue sections by using Leica BondRX autostainer (Leica). Human tonsil section was used as positive staining control, and the staining condition without primary antibodies was used as a negative control. The mIF optimization was performed as described previously (24). The same primary antibodies were used as described above for IHC, and Opal 7-color Automation IHC kit (Akoya Biosciences) was used for mIF staining. Each marker was assigned Opal fluorophores (Akoya Biosciences) as indicated in **Supplemental Table 1**, and DAPI was used for staining nuclei. The stained slides were scanned with Vectra 3 quantitative pathology imaging system (Akoya Biosciences), using unmixed signals on inForm software (Akoya Biosciences). Imaging analysis was performed on QuPath (25) to acquire cell segmentation data to locate each nucleated cell with each marker’s intensity and the classification of each cell type. Acquired cell segmentation data were used to measure distances between cells with R Script.

### Statistical analysis

We used Excel (Microsoft) and Prism 9 (GraphPad Software, RRID : SCR_002798) for graph generation and statistical analysis. Data are expressed as mean ± SEM where appropriate. High versus low levels of marker expression scores by IHC, flow cytometry, and IF were defined by the median. Differences between two groups were analyzed using paired or unpaired Student’s t-test, as appropriate, for parametric data. For analysis of three or more groups, one-way analysis of variance (ANOVA) tests was performed with Tukey’s or Dunnett’s post-test as appropriate. Results were considered statistically significant when P ≤ 0.05. Kaplan-Meier analyses and log-rank (Mantel-Cox) test were used to determine survival differences between groups. Correlations between two continuous variables were performed with Pearson correlation tests.

## Results

### Presence of NKp46+ NK cells in the sarcoma TME is associated with superior overall survival by immunohistochemistry

**Figure 1:**
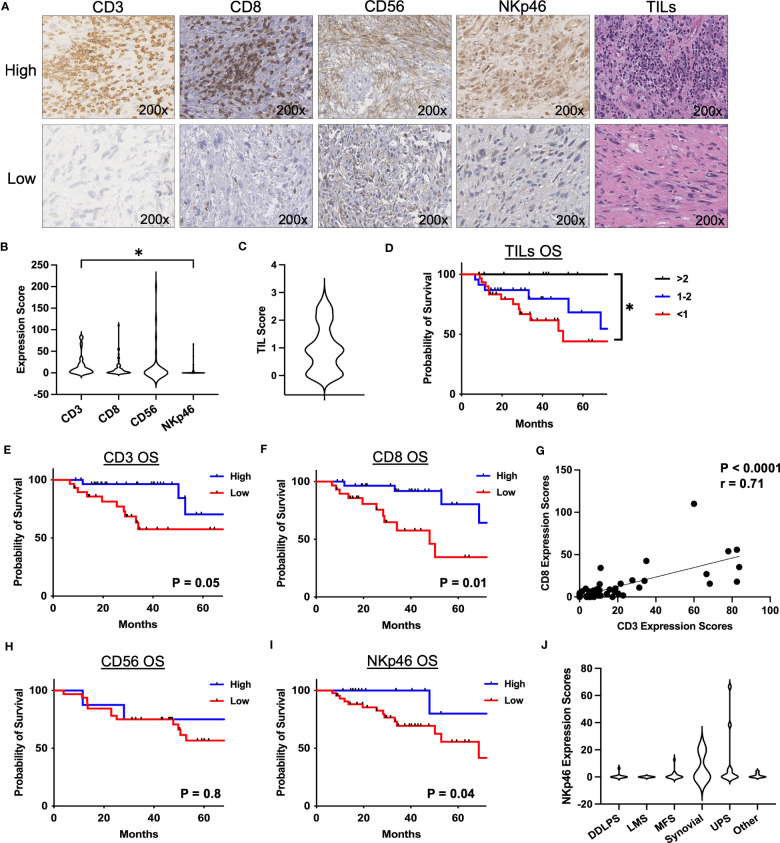
Presence of NKp46+ NK cells in the TME are associated with superior overall survival in STS. **(A)** Representative immunohistochemical photomicrographs of high and low staining for CD3, CD8, CD56, NKp46, and representative H&E staining for tumor infiltrating lymphocytes (TIL). **(B)** Distribution of mean expression scores for CD3, CD8, CD56, and NKp46 in the cohort (N=100). **(C)** Distribution of mean TIL scores. **(D)** Kaplan-Meier analysis of overall survival (OS) stratified by TIL scores showing survival benefit with higher TILs. **(E, F)** Kaplan-Meier analysis of OS stratified by median **(E)** CD3 and **(F)** CD8 expression scores. **(G)** Strong positive correlation of CD3 and CD8 expression scores (P<0.0001, r=0.71). **(H)** CD56 stratified by median expression score does not show an association with OS by Kaplan-Meier analysis. **(I)** Kaplan-Meier analysis of OS stratified by median NKp46 shows superior OS in patients with high NKp46 expression scores (P=0.04). **(J)** Distribution of NKp46 expression scores by histologic subtypes. DDPLS, dedifferentiated liposarcoma; LMS, leiomyosarcoma; TIL, tumor infiltrating lymphocyte; UPS, undifferentiated pleomorphic sarcoma; *, P<0.05.

As depicted in **Table 1**, our cohort included 130 STS patients undergoing surgery. 90 (69.3%) patients received preoperative radiotherapy. Overall, the cohort comprised locally advanced patients with mean tumor size of 12.8 cm (range 0.9 – 38.2), and 99 (76.2%) patients were classified as AJCC stage III. In order to assess the frequency and prognostic significance of established lymphocyte subsets, we stained TMA specimens from 100 patients (**Supplemental Table 2**) for the presence of TILs as well as CD3, CD8, CD56, and NKp46 subsets. Representative photomicrographs are shown in **Figure 1A**. Overall, expression of these immune markers was low, but with broad ranges (**Figure 1B**). Median H-score for CD3 was 6.6 (range 0-84), CD8 was 3.7 (range 0-110), CD56 was 0 (range 0-200), and NKp46 was 0 (0–67). Notably, the expression of CD3 was significantly higher than NKp46 (P=0.02). We then analyzed overall TIL scores, observing the majority of patients had low TILs with median score of 0.92 (range 0-3), consistent with prior studies (26) (**Figure 1C**). TIL scores >2 were associated with significantly improved survival (P=0.03) (**Figure 1D**). We then examined the prognostic significance of individual TIL subsets. As shown in **Figure 1E**, we observed superior OS among patients with CD3 expression above the median (P=0.05). OS was also higher among patients with high CD8 expression (P=0.01) (**Figure 1F**). As shown in **Figure 1G**, we observed a strong positive correlation between CD3 and CD8 expression scores (P<0.0001, r=0.71). We then examined the prognostic significance of NK markers, observing that differences in CD56 expression were not associated with differences in OS for either high or low values (P=0.8) (**Figure 1H**). In contrast, NKp46 expression was linked with prognosis with higher NKp46 expression associated with improved OS (P=0.04) (**Figure 1I**). Notably however, the expression of CD56 and NKp46 revealed a positive correlation with a P-value of 0.05 and an r of 0.71 (data not shown). Subgroup sizes for **Figures 1D–F, H, I** are available in **Supplemental Table 3**. Given the heterogeneity of STS types (27), we also evaluated NKp46 expression by histology, observing no significant differences among the most common subtypes (**Figure 1J**) despite the highest numerical staining in UPS tumors. Taken together, these data demonstrate NK and T cell numbers are generally low in STS, but carry prognostic significance, with differences in NK subsets either because of biological factors related to differences in CD56^+^ versus NKp46^+^ NK cells or technical factors related to IHC.

**Table 1:** Patient cohort and clinicopathologic characteristics.

Characteristics		Number (N=130)	%
Sex	Male Female	53 77	40.8% 59.2%
Age at diagnosis, (mean ± SD)		60.2 ± 17.6	
Maximal tumor size, cm, (mean ± SD)		12.8 ± 8.4	
Tumor site	Extremity Retroperitoneal Trunk Head and neck	83 28 18 1	63.9% 21.5% 13.8% 0.8%
Histology	Undifferentiated pleomorphic sarcoma Liposarcoma^a^ Myxofibrosarcoma Synovial sarcoma Leiomyosarcoma Other^b^	36 28 25 13 10 18	27.7% 21.5% 19.2% 10.0% 7.7% 13.8%
Tumor grade	High Intermediate Low	112 6 12	86.2% 4.6% 9.2%
Neoadjuvant therapy	Radiotherapy Upfront surgery Chemoradiation Chemotherapy only	72 35 18 5	55.4% 26.9% 13.9% 3.8%
Progression to metastases		61	46.9%
Vital status	Alive without evidence of disease Alive with disease Died	50 40 40	38.4% 30.8% 30.8%

a Includes 18 dedifferentiated liposarcoma, 6 myxoid liposarcoma, 2 well-differentiated liposarcoma, and 2 pleomorphic liposarcoma.

b Includes 3 rhabdomyosarcoma, 3 malignant peripheral nerve sheath tumor, 3 Ewing Family of tumors, 2 myxoid chondrosarcoma, 2 epithelioid sarcoma, 2 angiosarcoma, 1 fibromyxoid sarcoma, 1 solitary fibrous tumor, and 1 desmoplastic small round cell tumor.

### *CD56^dim^ NK* cells predominate in the sarcoma TME and are associated with a survival benefit

**Figure 2:**
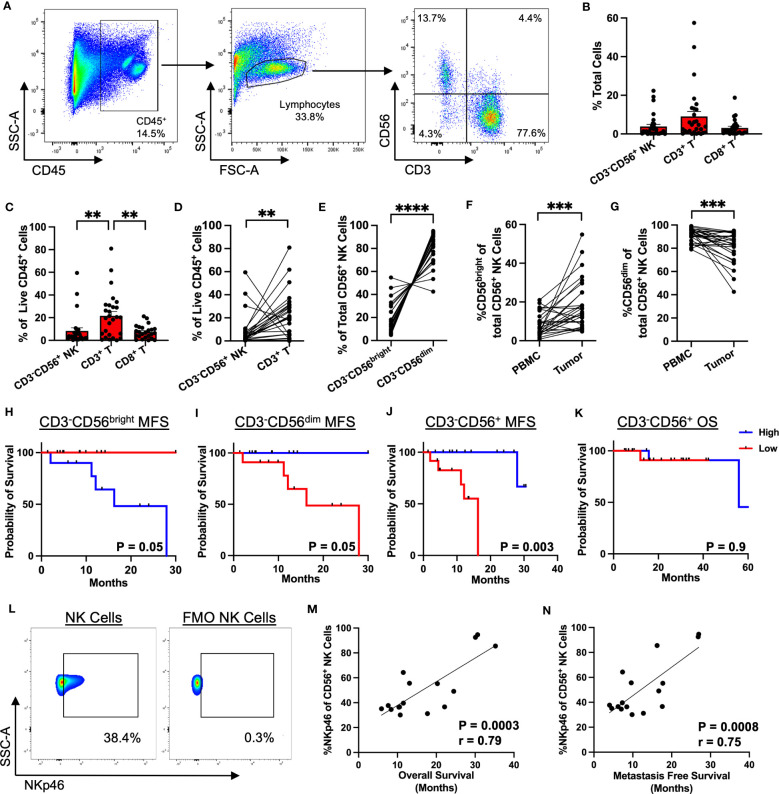
CD56^dim^ NK cells predominate in the TME with prognostic implications. **(A)** Representative flow cytometry showing gating strategy for identification of NK and T cells in STS TME. **(B)** Composition of the tumor immune infiltrate as a percent of total cells, showing CD3^-^CD56^+^ NK, CD3^+^ T, and CD8^+^ T cells. **(C)** Proportion of CD3^-^CD56^+^ NK, CD3^+^ T, and CD8^+^ T cells as a percent of live CD45^+^ cells. **(D)** Paired analysis of CD3^-^CD56^+^ NK and CD3^+^ T cells as a percent of live CD45^+^ cells (P=0.005). **(E)** Paired analysis of CD56^bright^ and CD56^dim^ intratumoral NK cells as a percent of total CD56^+^ cells (P<0.0001). **(F, G)** Paired analysis of **(F)** %CD56^bright^ and **(G)** %CD56^dim^ of total NK cells in peripheral blood and tumor. **(H, I)** Kaplan-Meier analysis of metastasis-free survival (MFS) stratified by median **(H)** CD3^-^CD56^bright^ and **(I)** CD3^-^CD56^dim^ frequencies. **(J, K)** Kaplan-Meier analysis of **(J)** MFS and **(K)** overall survival (OS) stratified by median CD3^-^CD56^+^ frequencies, showing MFS benefit with high CD3^-^CD56^+^ frequencies but no difference in OS. **(L)** Representative flow cytometry gating showing expression of NKp46 in the CD56^+^ NK cell population (left) and control FMO staining (right). **(M)** Strong positive correlation of %NKp46 of CD56^+^ NK cells with OS (P=0.0003, r=0.79). **(N)** Strong positive correlation of %NKp46 of CD56^+^ NK cells with MFS (P=0.0008, r=0.75). PBMC, peripheral blood mononuclear cell; **, P<0.01; ***, P<0.001, ****, P<0.0001; N=46.

Given the prognostic significance of NKp46^+^ NK cells and the limitations of single color IHC, we sought to further analyze the intratumoral NK cell phenotype and its impact on survival in STS using flow cytometry. Among our cohort of 130 patients, samples were prospectively collected for flow cytometry analysis for 46 patients (**Supplemental Table 2**). **Figure 2A** shows representative flow cytometry gating from STS tumor tissue obtained at the time of surgery. The frequency of immune infiltrates as a percent of total cells obtained from the surgical specimen is shown in **Figure 2B**, with generally low NK and T cells with some variability, consistent with our IHC data. CD3^-^CD56^+^ NK cells comprised 3.8% of total live cells (range 0.03-22.3%), CD3^+^ T cells comprised 9.0% (range 0.1-57.5%), and CD8^+^ T cells comprised 3.1% (range 0.03-18.8%). As shown in **Figure 2C**, CD3^+^ T cells were the most abundant intratumor lymphocyte (21.7% ± 3.7%), followed by CD3^-^CD56^+^ NK (8.4% ± 2.6%) (P=0.004) and CD8^+^ T cells (7.4% ± 1.1%) (P=0.003). We then performed paired analysis of intratumoral and peripheral blood immune frequencies. Intratumoral CD3^-^CD56^+^ NK and CD3^+^ T cells as percent of live CD45^+^ cells demonstrated an inverse relationship with higher NK cells corresponding to significantly lower T cells in the tumor and vice versa (P=0.005, **Figure 2D**).We also observed a comparable inverse relationship among paired samples of NKp46 and CD3 expression scores by IHC (**Supplemental Figure 1**). Paired analysis of CD3^-^CD56^bright^ and CD3^-^CD56^dim^ cells as a percentage of total CD56^+^ cells revealed significantly greater CD56^dim^ frequencies in both tumor and blood (P<0.0001) (**Figures 2E–G**). Interestingly, although of CD56^bright^ NK cells were enriched in STS tumors and the intratumoral frequency higher compared to blood (P=0.0006) (**Figure 2F**), CD56^dim^ NK cells remained the significant majority of intratumoral NK cells (80.9% ± 2.6%) (**Figure 2G**). Notably, higher levels of CD3^-^CD56^bright^ NK cells in the tumor, when stratified by the median percentages, demonstrated significantly worse MFS compared to lower levels of CD3^-^CD56^bright^ NK cells (P=0.05) (**Figure 2H**). Conversely, higher levels of intratumoral CD3^-^CD56^dim^ NK cells were associated with superior MFS compared to lower levels (P=0.05) (**Figure 2I**), suggesting that the specific subset of NK cells does have a significant impact on the biology and prognosis in STS. Overall, the frequency of intratumoral CD3^-^CD56^+^ NK cells was also associated with significantly greater MFS (**Figure 2J**), although we observed no difference in OS when stratified by high and low overall CD3^-^CD56^+^ NK frequencies, perhaps reflecting the shorter duration of median follow up of our flow cytometry cohort (30.7 ± 2.6 months) (**Figure 2K**). We then evaluated expression of the activation marker NKp46 on CD56^+^ NK cells (**Figure 2L**) which demonstrated a strong positive correlation with both OS (P=0.0003, r=0.79) (**Figure 2M**) and MFS (P=0.0008, r=0.75) (**Figure 2N**). Similarly, we analyzed the correlations of NKp46 median fluorescence intensity (MFI) of CD3^-^CD56^+^ NK cells in blood and tumor. No differences in NKp46 MFI between blood and tumor were noted, but we observed significant positive correlations of NKp46 MFI in blood and tumor with both OS and MFS (**Supplemental Figure 2**). However, our sample size of patients with flow data on NKp46^+^ NK cell expression was limited (N=16), thereby precluding meaningful Kaplan-Meier survival analyses for this subset. Given the importance of histology in governing outcomes in STS, we analyzed the breakdown of intratumoral CD56^bright/dim^ NK cells by histology (**Supplemental Figure 3**). Overall, CD56^dim^ NK cells comprised the vast majority of intratumoral STS NK cells with no significant differences among histologies in our cohort. Taken together, these data demonstrate that CD56^dim^ NK cells predominate in the STS tumor microenvironment with a more favorable impact on outcomes among CD56^dim^ NK cells than CD56^bright^.

### Spatial analysis of STS immune infiltrate by immunofluorescence demonstrates distinct localization of NK and T cells

**Figure 3:**
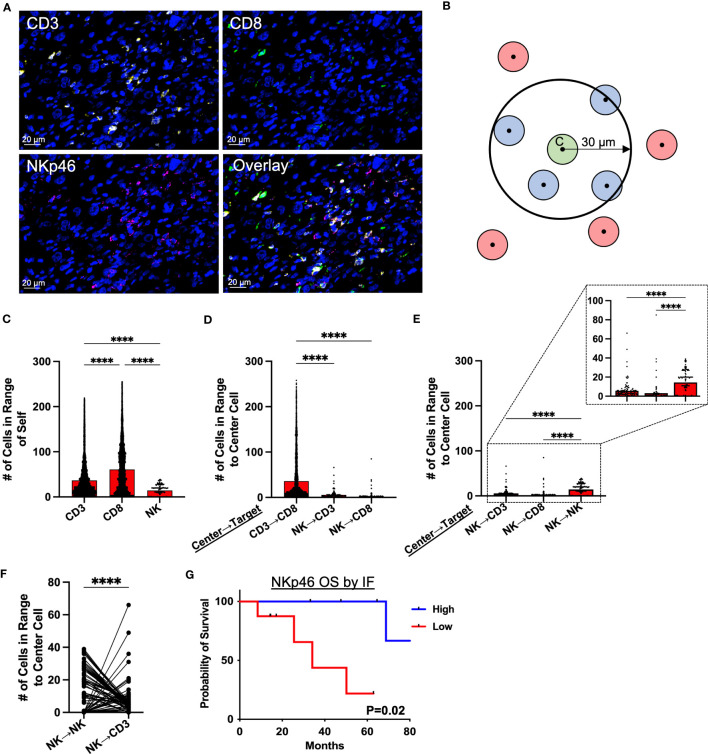
Spatial analysis of STS immune infiltrate by immunofluorescence demonstrates distinct localization of NK and T cells. **(A)** Representative immunofluorescence (IF) photomicrographs of CD3 (yellow), CD8 (green), NKp46 (pink), and overlay of the three markers in soft tissue sarcoma (STS) tumor microenvironment (TME), with nuclear staining by DAPI in blue. **(B)** Graphic illustrating spatial analysis of target cells (blue) within 30μm radius of a center cell (green). Cells out of radius (red) were not considered in-range. **(C)** Number of CD3^+^ T, CD8^+^ T, and NKp46^+^ NK cells within 30μm radius of index effector cell of interest. **(D)** Number of CD3^+^ T, CD8^+^ T, and NKp46^+^ NK cells in 30μm radius to center cells (CD3^+^ T and NKp46^+^ NK cells), showing significantly greater number of CD3^+^ T and CD8^+^ T cells in range to CD3^+^ T center cells than to NKp46^+^ NK cells. **(E)** Number of CD3^+^ T, CD8^+^ T, and NKp46^+^ NK cells in 30μm radius to NKp46^+^ NK center cells, showing significant clustering of NKp46^+^ NK with other NKp46^+^ NK compared to T cells, with a further zoomed-in view indicated by the dotted boxes and lines. **(F)** Paired analysis of the number of NK and CD3^+^ T cells in range, showing an inverse relationship between the presence of NK and CD3^+^ T cells around a center NK cell. **(G)** Kaplan-Meier analysis of OS stratified by median NKp46 expression on IF showing superior OS in patients with high NKp46 expression. ****, P<0.0001; N=71.

Given the potential for NK cells to respond to different activating and inhibitory signals than T cells (in particular MHC-I), we then sought to characterize the relative spatial organization of our effector cells of interest (CD3, CD8, and NKp46) in the STS TME from 71 patients (**Figure 3A**; **Supplemental Table 2**) to investigate the implications of potential local interactions of immune subsets within the TME. As illustrated by the graphic in **Figure 3B**, we used cell segmentation data derived from QuPath imaging analyses to measure spatial cell-cell associations of target cells (blue) within a 30μm radius of an index center cell (green) in the sarcoma TME. We first evaluated the spatial localization of each marker with respect to itself (**Figure 3C**), observing that CD8^+^ T cells had the greatest clustering with themselves, with an average of 61 CD8^+^ T cells (range 0-255) around each center CD8^+^ T cell, followed by CD3^+^ T cells with an average of 37 CD3^+^ T cells (range 0-219) around each center CD3^+^ T cell (P<0.0001). NK cells, as determined by NKp46 expression, had an average of 14 NK cells (range 0-39) clustered around each center NK cell, though overall NK cell infiltration was lower compared to that of T cells (P<0.0001). We then investigated the relative spatial localization of T and NK cells with respect to these counterpart cells (**Figure 3D**). CD8^+^ T cells showed the greatest clustering around CD3^+^ T cells, with an average of 36 CD8^+^ T (range 0-258) per CD3^+^ T cell, and this clustering was markedly higher (P<0.0001) than that of either CD3^+^ T or CD8^+^ T cells localizing around center NK cells, with an average of 6 (range 0-66) CD3^+^ T cells and 3 (range 0-85) CD8^+^ T per center NK cell, respectively (**Figure 3D**). Comparing the number of CD3^+^ T and CD8^+^ T cells to the number of NK cells in 30μm radius to NK center cells, we observed significantly greater clustering of NK cells with other NK cells than with CD3^+^ T (P<0.0001) and CD8^+^ T cells (P<0.0001) (**Figure 3E**). To further examine the spatial relationship between NK and T cells, we performed a paired analysis of the number of NK and CD3^+^ T cells in range of center NK cells, which demonstrated a significantly inverse relationship between the two cell types (**Figure 3F**). When NK cells were highly clustered around a center NK cell, the same center NK cell had low number of CD3^+^ T cells in range, and when higher number of CD3^+^ T cells were present, the same center NK cell had low clustering with other NK cells. With an average of 14 (range 0-39) NK cells as compared to 6 (range 0-66) CD3^+^ T cells within range of the center NK cells, this analysis further emphasizes that NK cells have greater spatial localization with other NK cells compared to CD3^+^ T (P<0.0001). Similar to our IHC data (**Figure 1I**), greater NKp46 expression by immunofluorescence, stratified by above or below the median, was associated with statistically superior OS (**Figure 3G**; **Supplemental Table 3**) and a similar overall trend in MFS (**Supplemental Figure 4**), further reinforcing the prognostic significance of NKp46^+^ NK cells in STS. Taken together, these data demonstrate that NK cells and T cells have distinct spatial localization patterns in the sarcoma TME with NK cells appearing to preferentially localize among other NK cells with sparse neighboring CD3^+^ T and CD8^+^ T cells and vice versa.

### Impact of neoadjuvant radiotherapy and chemotherapy on intratumoral NK and T cell clustering

**Figure 4:**
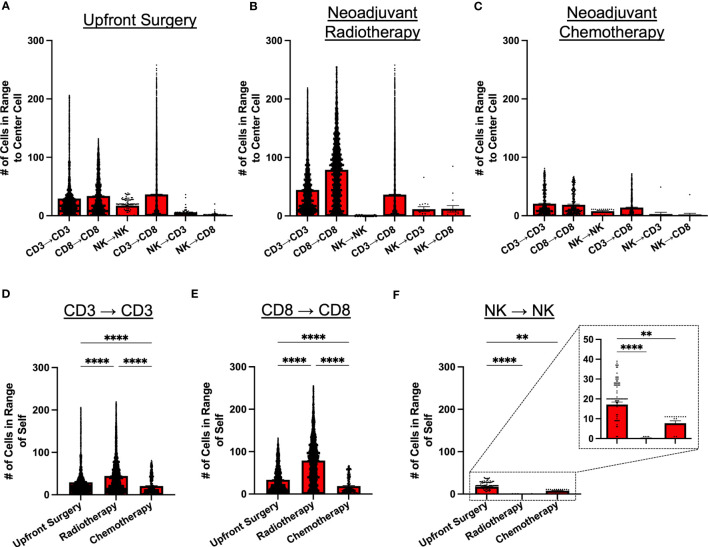
Neoadjuvant radiotherapy and chemotherapy have differential effects on intratumoral immune cell clustering. **(A–C)** Number of CD3^+^ T, CD8^+^ T, and NKp46^+^ NK cells within 30μm radius of effector cell of interest in patients that received **(A)** upfront surgery, **(B)** neoadjuvant radiotherapy, and **(C)** neoadjuvant chemotherapy. **(D)** Number of CD3^+^ T cells in range of other CD3^+^ T cells by treatment group, showing the highest clustering of CD3^+^ T cells in the radiotherapy group. **(E)** Number of CD8^+^ T cells in range of other CD8^+^ T cells by treatment group, showing the highest clustering of CD8^+^ T cells in the radiotherapy group. **(F)** Number of NKp46^+^ NK cells in range of other NKp46^+^ NK cells by treatment group, showing significantly lower clustering in the radiotherapy and chemotherapy groups compared to upfront surgery, with a further zoomed-in view indicated by the dotted boxes and lines. ****, P<0.0001; **, P<0.01.

Given the potential for variability in treatment sequencing for STS patients with localized disease, we analyzed patients according to type of initial cancer therapy, comparing intratumoral immune cell spatial localization in patients receiving upfront surgery, neoadjuvant radiotherapy (RT), and neoadjuvant chemotherapy (either as monotherapy or as chemoradiation) (**Figures 4A–C**, respectively). Overall, we observed similar patterns of cell clustering across treatment types, with CD3^+^ and CD8^+^ T cells showing the greatest clustering with themselves and each other, while NK cell clustering among other NK cells or with CD3^+^ and CD8^+^ T cells was significantly lower. However, despite these broad similarities in NK and T cell clustering, we did observe some significant differences with potential clinical significance. Neoadjuvant radiotherapy, for example, was associated with overall greater cell clustering of CD3^+^ and CD8^+^ T cells with each other, with significantly lower clustering of NK cells with other NK cells (**Figures 4B, F**). We also observed that overall immune cell clustering was lower among patients receiving neoadjuvant chemotherapy, though the general pattern of immune cell clustering remained constant (**Figure 4C**). Further statistical analyses for **Figures 4A–C** are available in **Supplemental Table 4**. We evaluated spatial relationships of immune cells with themselves by treatment groups. We observed significantly greater clustering of CD3^+^ T cells with other CD3^+^ T cells and CD8^+^ T cells with other CD8^+^ T cells in patients receiving neoadjuvant RT compared to upfront surgery and chemotherapy (**Figures 4D, E**, respectively). We also observed significantly lower clustering of NK cells with other NK cells (**Figure 4F**), suggesting differential effects of neoadjuvant RT on the lymphocyte population in the STS TME. Finally, patients receiving neoadjuvant chemotherapy had significantly less clustering of all 3 lymphocyte populations (**Figures 4D–F**), potentially due to the cytotoxic effects of chemotherapy, also suggesting differential effects of neoadjuvant chemotherapy on NK and T cell infiltrates in the STS TME.

### MHC-I^+^ cells show greater clustering with T cells than NK cells by spatial analysis

**Figure 5:**
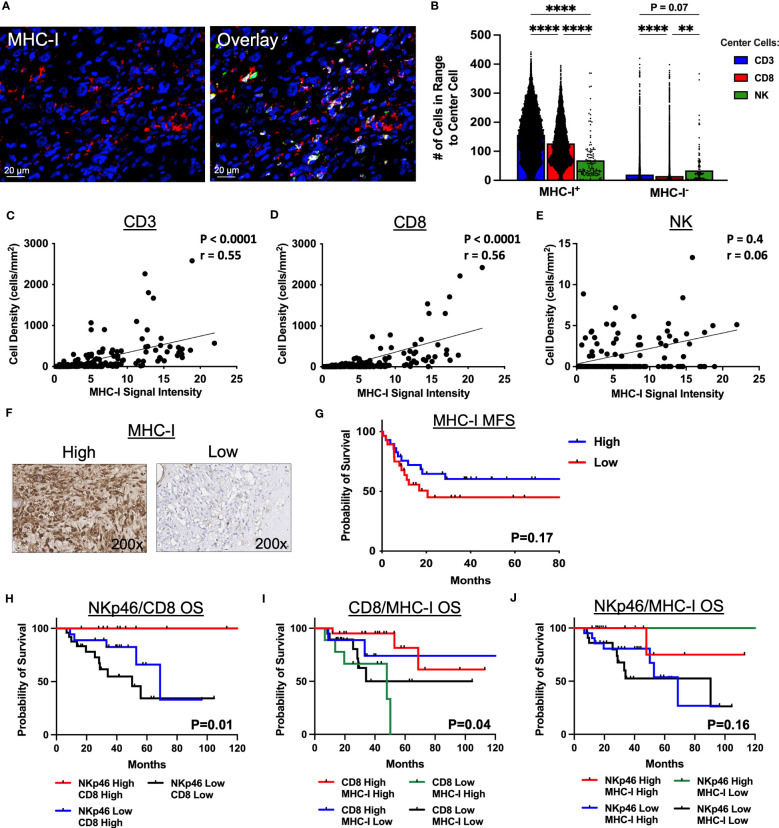
MHC-I^+^ cells show greater clustering with T cells than NK cells by immunofluorescence spatial analysis. **(A)** Representative immunofluorescence photomicrographs of MHC-I and overlay with CD3 (yellow), CD8 (green), and NKp46 (pink). **(B)** Number of CD3^+^ T, CD8^+^ T, and NKp46^+^ NK cells in 30μm radius to MHC-I^+^ and MHC-I^-^ cells. **(C–E)** MHC-I expression correlates significantly with **(C)** CD3^+^ T cell density (P<0.0001, r=0.55) and **(D)** CD8^+^ T cell density (P<0.0001, r=0.56), but not with **(E)** NK cell density (P=0.4, r=0.06). **(F)** Representative immunohistochemical photomicrographs of high and low staining for MHC-I. **(G)** Kaplan-Meier analysis of metastasis-free survival (MFS) stratified by median MHC-I expression score showing improved MFS in patients with high MHC-I expression scores which is not statistically significant. **(H–J)** Kaplan-Meier analyses of overall survival (OS) stratified by high and low expression scores determined by the median of **(H)** NKp46 and CD8 (P=0.01), **(I)** CD8 and MHC-I (P=0.04), and **(J)** NKp46 and MHC-I (P=0.16). ****, P<0.0001; **, P<0.01; N=71 for immunofluorescence; N=100 for immunohistochemistry.

Given the distinct spatial localization and clustering of NK cells and T cells in the STS TME, we sought to evaluate the impact of MHC-I expression on these results considering the principles of the “missing-self” hypothesis and the role of MHC-I expression in both NK and T cell target recognition. **Figure 5A** depicts representative photomicrographs of MHC-I expression by IF with overlay of CD3, CD8, and NKp46. As shown in **Figure 5B**, clustering of CD3^+^ and CD8^+^ T cells was significantly higher around MHC-I^+^ cells compared to NKp46^+^ NK cells (P<0.0001 for both). In contrast, although clustering around MHC-I^-^ cells was numerically lower among all 3 effector cells (CD3^+^, CD8^+^, NKp46^+^) compared to MHC-I^+^ cells, NK cells demonstrated significantly greater clustering around MHC-I^-^ cells than CD8^+^ T cells and a trend for significantly greater clustering compared to CD3^+^ T cells (P=0.07). We then analyzed the correlation of MHC-I expression with the cell density of CD3^+^ T cells, CD8^+^ T, and NKp46^+^ NK cells (**Figures 5C–E**, respectively), observing a moderate positive correlation between both CD3^+^ T cells and MHC-I^+^ cells (P<0.0001, r=0.55) and CD8^+^ T cells and MHC-I+ cells (P<0.0001, r=0.056). In contrast, we observed no association between MHC-I expression and NKp46^+^ NK cell density (P=0.4, r=0.06). To evaluate MHC-I further, we analyzed MHC-I expression by IHC (**Figure 5F**) and noted improved MFS among patients with MHC-I expression above the median, but this was not statistically significant (**Figure 5G**). Lastly, we evaluated survival outcomes using IHC marker expression of NKp46, CD8, and MHC-I stratified into 4 groups by two of these three markers, with further subgroup sizes and statistical analyses in **Supplemental Tables 3**, **5**, respectively. As shown in **Figure 5H**, high expression of both NKp46 and CD8 showed significantly greater OS compared to NKp46 low/CD8 high and NKp46 low/CD8 low, respectively (P=0.01) with the NKp46 high/CD8 low subgroup having insufficient number of patients for analysis. When analyzing CD8 expression as a function of MHC-I, we observed that patients with high CD8 and either high or low MHC-I expression had improved OS compared to patients with low CD8 expression and either high or low MHC-I expression (P=0.04) (**Figure 5I**). Finally, when analyzing NKp46 expression as a function of MHC-I, we observed that patients with high expression of NKp46 and low expression of MHC-I had 100% event-free survival, compared to the other subgroups, although this result was not statistically significant (P=0.16) (**Figure 5J**). Notably, %CD69^+^ NK cells, a recognized marker of activation, were higher in patients with low MHC-I expression compared to high MHC-I expression, though this did not reach statistical significance (P=0.07) (**Supplemental Figure 5**). Collectively, these results indicate that MHC-I^+^ cells preferentially localize among CD3^+^ T and CD8^+^ T cells in contrast to NK cells, reinforcing distinct localization patterns of NK and T cells in the STS TME, and that the pattern of NKp46, CD8, and MHC-I expression in the sarcoma TME has prognostic significance. Although spatially distinct, our data also suggest a synergistic relationship between NKp46^+^ NK cells and CD8^+^ T cells in the TME.

## Discussion

Recent breakthroughs in cancer immunotherapy have focused on amplifying T-cell-mediated pathways. However, successes with immunotherapy in STS treatment have been limited, emphasizing the unmet need for the development of novel immunotherapies for STS. Although NK cells are exciting as a cancer therapeutic and have been shown to be prognostic in multiple solid tumor types (28, 29) including STS, successes to date have been scant and a better understanding on the phenotype and function of intratumoral NK cells is needed. Using multiple assays, we demonstrate the prognostic significance of NK cells in STS. Despite overall low intratumoral immune cell infiltration, higher levels of NK cells provide a survival benefit in STS patients across multiple readouts. Similar findings have been demonstrated in other solid tumors, such as gastric, colorectal, lung, and head and neck cancers (30, 31), thus augmentation of NK cell numbers and effector functions remain a promising approach to improve clinical outcomes in solid tumors, such as STS.

However, the NK cell population remains diverse, consisting of various subsets likely with differing roles in the TME (32). Here, we showed by IHC that high expression of NKp46, a recognized marker of NK cell activation (33), is associated with improved OS, while CD56 expression by IHC does not correlate with better survival outcomes, suggesting differences in NK effector functions between NKp46^+^ and CD56^+^ NK cells. Our analysis of CD3^-^CD56^+^ NK cells by flow cytometry showed that greater infiltration of CD3^-^CD56^+^ NK cells is associated with improved metastasis-free survival in STS, but not with superior OS. When stratified by CD56^bright^ versus CD56^dim^ sub-populations, we observed that CD56^dim^ NK cells, the more cytotoxic subset compared to its CD56^bright^ counterpart, were the predominant intratumoral NK cell in STS and were associated with improved MFS, an association that has also been observed in squamous cell head and neck cancers but not in invasive bladder cancer (34, 35). Furthermore, data by Mukherjee et al. linked high infiltration of CD56^bright^ NK cells with improved survival in invasive bladder cancer (35) while our data, in concordance with studies in Ewing sarcoma, neuroblastoma, and thyroid papillary cancer, showed that high levels of CD56^bright^ NK cells are associated with worse prognosis (36–38). On balance, CD56^bright^ versus CD56^dim^ NK cell subsets appear to play different roles in the TME, although there is a lack of consensus regarding subset effects on outcomes. Ultimately, our data support a survival benefit for CD56^dim^ NK cells and an unfavorable effect for CD56^bright^ NK cells in STS. Moreover, our finding of greater CD56^dim^ NK cells in the STS TME is counter to the findings of Carrega et al. and underscores the importance of better delineation of tumor-reactive versus dysfunctional NK cells in the TME to help unlock the potential of NK cells in solid cancers (39).

Although prior studies have characterized the spatial organization of NK cell and T cell subsets in pancreatic, colorectal, and lung cancers, the emphasis of these studies was on the proximity of immune populations to various tumor compartments such as the invasive margin, the stroma, or the tumor core (40–43). To our knowledge, a spatial analysis of NK and T cell subsets in STS and how they cluster with each other and with MHC-I^+^ cells has not been investigated to date. Our spatial analyses of immune infiltrates in STS, including NKp46^+^ NK cells, demonstrated that NK cells are increasingly clustered amongst other NK cells with sparse clustering to CD3^+^ T and CD8^+^ T cells. Additionally, we revealed an inverse relationship between NK and T cell localizations, indicating that NK cells and T cells have distinct spatial localization patterns in the STS TME. This relationship likely stems, at least in part, due to the different effects of MHC-I expression on NK and T cells in the TME. MHC-I acts as an inhibitory signal for NK cells and MHC-I downregulation contributes to the activation of cytotoxic NK cell effector functions. In contrast, CD3^+^ and CD8^+^ T cells respond to antigen presentation by MHC-I, and MHC-I downregulation has been associated with T cell suppression and resistance to ICI (44).

Further analysis of the spatial localization of MHC-I^+^ and MHC-I^-^ cells with respect to immune infiltrate revealed high clustering of CD3^+^ T and CD8^+^ T cells with MHC-I^+^ cells, while NK cells showed greater clustering with MHC-I^-^ cells than either T cell subset. This finding was further strengthened with significant correlations between CD3^+^ T and CD8^+^ T cells with MHC-I, while NK cells did not show an association. Downregulation of MHC-I in multiple cancers, including STS, is a well-established mechanism of evasion of the immune system, especially that of cytotoxic CD8^+^ T cells (45–48). However, these effects may have regional roles in the TME. Our data suggest that overall, the presence of CD8^+^ T cells and NK cells in the STS TME are each beneficial for clinical outcomes, but it has not been well defined whether NK and T cells cooperate in anti-tumor responses or substitute for one another depending on the cellular and molecular signals. We observed improved survival outcomes of patients with high levels of either NKp46 or CD8 cells in the sarcoma TME, but also an inverse relationship with NK cells being more prevalent when T cells were low and T cells more prevalent when NK cells were low. Ultimately, these data reinforce the impression that that NK cells and CD8^+^ T cells play separate but interconnected roles in the STS TME, with evidence for micro-clustering of NK around other NK cells and CD8^+^ T cells around other CD8^+^ T cells and around MHC-I^-^ and MHC-I^+^ cells, respectively, but not around each other. A similar phenomenon was described where CD8^+^ T cells were observed attacking MHC-I^+^ antigen presenting cells, while NK cells attacked cells with downregulated MHC-I expression, in their respective microregions (49). Overall, spatial analysis via multiplex immunofluorescence has allowed us to take a step further than single color IHC allows, consequently shed light on possible interactions within the TME, their implications on patient outcomes, and the potential for biomarkers of prognosis and immunotherapy treatment selection.

Despite the interesting findings described here within, it is important to recognize the limitations of our work. Although our overall sample size included 130 patients, prospective tumor collection for flow cytometry analyses was limited to a cohort of 46 patients, decreasing the power of our conclusions. Given the nature of prospective studies and the relatively shorter follow-up time for prospective patient cohorts, fewer events were present for survival analysis from this subset regarding the impact of CD56^dim^ NK cell infiltration on overall survival. Our analysis of co-expression of NKp46^+^ by CD56^+^ NK cells was similarly limited by sample size and overall follow up time. Nevertheless, considering the rare nature of STS, prospective enrollment of 46 patients for analysis compares favorably to other studies. Similarly, although we recognize that STS consists of over 70 histologic subtypes (50) with inherent differences between subtypes in the immune TME, subtype specific analyses are challenging even for multi-institutional studies given the rare nature of STS, and we did not observe significant differences in NK infiltration among the various histologies in our cohort.

In summary, we identified both NKp46 and CD56^dim^ as markers of intratumoral NK cells associated with a favorable prognosis in STS, highlighting the clinical relevance of heterogeneous subsets of intratumoral NK cells. Localization analyses revealed distinct micro-environments of NK cells and T cells primarily clustered by the presence or absence of MHC-I expression, reinforcing the importance of this fundamental immune recognition complex in regulating anti-tumor responses. Ultimately, a better understanding of the complex factors regulating NK infiltration, phenotype, and function in the STS TME could lead to more effective immunotherapies, especially in “cold” tumors like STS, which have historically demonstrated poor responses to T-cell-dependent immunotherapies.

## Data availability statement

The raw data supporting the conclusions of this article will be made available by the authors, without undue reservation.

## Ethics statement

The studies involving human participants were reviewed and approved by University of California, Davis, Institutional Review Board. The patients/participants provided their written informed consent to participate in this study.

## Author contributions

SC, SJ, and RC designed the study. SC, CS, SJ, MD, KI, LF, CD, AG, HM, and RC conducted the experiments and collected the data. SC, CS, SJ, MD, AG, HM, and RC analyzed the data. SC and RC wrote the manuscript. All authors, including ML, AR, JP, ST, AM, and WM, provided critical reviews of the manuscript. All authors contributed to the article and approved the submitted version.
