# Supplementary material for: Intratumoral NKp46+ natural killer cells are spatially distanced from T and MHC-I+ cells with prognostic implications in soft tissue sarcoma
Source: Front Immunol. 2023 Jul 21;14:1230534. doi: 10.3389/fimmu.2023.1230534 (PMC10401426; doi:10.3389/fimmu.2023.1230534)
Supplement: Supplementary file 1 [file DataSheet_1.pdf]

Supplementary Material

Intratumoral NKp46+ Natural Killer Cells are Spatially Distanced from T and MHC-I+ Cells with Prognostic Implications in Soft Tissue Sarcoma

Sylvia M. Cruz[1], Cyrus J. Sholevar[1], Sean J. Judge[2], Morgan A. Darrow[3], Khurshid R. Iranpur[1], Lauren E. Farley[1], Marshall Lammers[1], Aryana M. Razmara[1], Cordelia Dunai[4], Alicia A. Gingrich[5], Julia Persky[1], Hidetoshi Mori[6], Steven W. Thorpe[7], Arta M. Monjazeb[8], William J. Murphy[4], Robert J. Canter[1]

[1]Division of Surgical Oncology, Department of Surgery, University of California Davis, Sacramento, CA, USA
[2]Department of Surgery, Memorial Sloan Kettering Cancer Center, New York, NY, USA
[3]Pathology and Laboratory Medicine, University of California Davis, Sacramento, CA, USA
[4]Department of Dermatology, University of California Davis, Sacramento, CA, USA
[5]Department of Surgical Oncology, MD Anderson Cancer Center, Houston, TX, USA
[6]Center for Immunology and Infectious Diseases, University of California Davis, Sacramento, CA, USA
[7]Orthopedic Surgery, University of California Davis, Sacramento, CA, USA
[8]Radiation Oncology, University of California Davis, Sacramento, CA, USA

*** Correspondence:**
Dr. Robert J. Canter
Professor of Surgery
Division of Surgical Oncology
University of California, Davis
4501 X Street, Suite 3010
Sacramento, CA 95817
Office: 916-734-5907
Email: rjcanter@ucdavis.edu

Supplemental Tables

Supplemental Table 1. Multiplex Immunofluorescence Antibodies and Opal Fluorophores

Staining Cycle	Target Molecule	Company	Clone	Antibody Dilution	Reaction Time for Primary Antibody	Blocking	Secondary Antibody	Reaction Time for Secondary Antibody	Opal Dye	Opal Dye Dilution	Reaction Time for Opal Dye	Stripping and Termination for Reaction
1	NKP46	Abcam	EPR22403-57	1:250	32min/RT	30min/RT	Opal Polymer HRP	10min/RT	620	1:100	10min/RT	ER1 20min/98°C
2	CD8	DAKO	clone C8/114B	1:100	32min/RT	10min/RT	Opal Polymer HRP	10min/RT	520	1:100	10min/RT	ER1 20min/98°C
3	CD3	Ventana	2GV6	1:2	32min/RT	10min/RT	Opal Polymer HRP	10min/RT	570	1:300	10min/RT	ER1 20min/98°C
4	MHC-I	Abcam	EMR8-5	1:3000	32min/RT	10min/RT	Opal Polymer HRP	10min/RT	650	1:200	10min/RT	ER1 20min/98°C

Supplemental Table 2. Patient cohort and clinicopathologic characteristics of 130 patients by methods of analysis

Characteristic		Immunohistochemistry (N=100)	Flow Cytometry (N=46)	Immunofluorescence (N=71)
Sex	Female	41	19	32
	Male	59	27	39
Age at diagnosis, (mean ± SD)		59.8 ± 18.2	62.2 ± 15.3	61.8 ± 17.2
Maximal tumor size, cm, (mean ± SD)		13.2 ± 8.3	13.4 ± 9.6	13.9 ± 9.9
Tumor site	Extremity	67	25	41
	Retroperitoneal	19	15	19
	Trunk	13	6	10
	Head and neck	1	0	1
Histology	Undifferentiated pleomorphic sarcoma	30	9	16
	Liposarcoma[a]	22	11	20
	Myxofibrosarcoma	18	11	16
	Synovial sarcoma	9	5	4
	Leiomyosarcoma	6	6	6
	Other[b]	15	4	9
Tumor grade	High	84	43	59
	Intermediate	5	1	1
	Low	11	2	11
Neoadjuvant therapy	Radiation monotherapy	53	28	40
	Upfront surgery	29	8	19
	Chemoradiation	13	10	9
	Chemotherapy only	5	0	3
Progression to metastases		53	15	39
Vital status	Alive without evidence of disease	37	20	23
	Alive with disease	35	18	24
	Died	28	8	24

[a]Includes IHC (12 dedifferentiated liposarcoma, 6 myxoid liposarcoma, 2 well-differentiated liposarcoma, and 2 pleomorphic liposarcoma), FC (10 dedifferentiated liposarcoma, 1well-differentiated liposarcoma), and IF (12 dedifferentiated liposarcoma, 5 myxoid liposarcoma, 2 well-differentiated liposarcoma, and 1 pleomorphic liposarcoma)

[b]Includes IHC (Includes 3 rhabdomyosarcoma, 3 malignant peripheral nerve sheath tumor, 3 Ewing Family of tumors, 2 myxoid chondrosarcoma, 2 epithelioid sarcoma, 2 angiosarcoma, 1 fibromyxoid sarcoma 1 solitary fibrous tumor, and 1 desmoplastic small round cell tumor), FC (1 rhabdomyosarcoma, 1 Ewing Family of tumors, 1 epithelioid sarcoma, and 1 desmoplastic small round cell tumor), and IF (1 rhabdomyosarcoma, 2 malignant peripheral nerve sheath tumor, 2 Ewing Family of tumors, 2 angiosarcoma, 1 solitary fibrous tumor, and 1 desmoplastic small round cell tumor). IHC, immunohistochemistry; FC, flow cytometry; IF, immunofluorescence.

Supplemental Table 3. Additional Subgroup Data

Figure	Group	Number of Patients
1D	>2	9
	1-2	23
	<1	32
1E	High	30
	Low	29
1F	High	30
	Low	29
1H	High	16
	Low	44
1I	High	9
	Low	32
3G	High	6
	Low	8
5G	High	30
	Low	30
5H	NKp46 High/CD8 High	12
	NKp46 Low/CD8 High	18
	NKp46 Low/CD8 Low	25
5I	CD8 High/MHC-I High	21
	CD8 High/MHC-I Low	9
	CD8 Low/MHC-I High	9
	CD8 Low/MHC-I Low	20
5J	NKp46 High/MHC-I High	9
	NKp46 High/MHC-I Low	7
	NKp46 Low/MHC-I High	21
	NKp46 Low/MHC-I Low	23

Supplemental Table 4. Statistical Analyses for Figure 4

Groups Compared	Figure 4A P-value	Figure 4B P-value	Figure 4C P-value
NK→NK vs. CD3→CD3	0.0339	0.0025	0.0827
NK→NK vs. CD8→CD8	0.0008	<0.0001	0.2149
NK→NK vs. NK→CD3	0.2778	0.9859	0.9814
NK→NK vs. NK→CD8	0.0860	0.9811	0.9608
NK→NK vs. CD3→CD8	<0.0001	0.0275	0.8012
CD3→CD3 vs. CD8→CD8	<0.0001	<0.0001	0.7267
CD3→CD3 vs. NK→CD3	<0.0001	0.0740	0.0037
CD3→CD3 vs. NK→CD8	<0.0001	0.0868	0.0018
CD3→CD3 vs. CD3→CD8	<0.0001	<0.0001	<0.0001
CD8→CD8 vs. NK→CD3	<0.0001	<0.0001	0.0162
CD8→CD8 vs. NK→CD8	<0.0001	<0.0001	0.0089
CD8→CD8 vs. CD3→CD8	0.0034	<0.0001	0.0047
NK→CD3 vs. NK→CD8	0.9949	>0.9999	>0.9999
NK→CD3 vs. CD3→CD8	<0.0001	0.3202	0.2143
NK→CD8 vs. CD3→CD8	<0.0001	0.3553	0.1448

Supplemental Table 5. Additional Statistical Analyses for Figure 5

Figure	Groups Compared	P-value
5H	NKp46 High/CD8 High vs. NKp46 Low/CD8 Low	0.0068
	NKp46 High/CD8 High vs. NKp46 Low/CD8 High	0.0461
	NKp46 Low/CD8 High vs. NKp46 Low/CD8 Low	0.1174
5I	CD8 High/MHC-I High vs. CD8 Low/MHC-I Low	0.0497
	CD8 High/MHC-I High vs. CD8 High/MHC-I Low	0.3867
	CD8 High/MHC-I High vs. CD8 Low/MHC-I High	0.0007
	CD8 High/MHC-I Low vs. CD8 Low/MHC-I High	0.2635
	CD8 High/MHC-I Low vs. CD8 Low/MHC-I Low	0.3925
	CD8 Low/MHC-I High vs. CD8 Low/MHC-I Low	0.4735
5J	NKp46 High/MHC-I High vs. NKp46 Low/MHC-I Low	0.0969
	NKp46 High/MHC-I High vs. NKp46 High/MHC-I Low	0.6171
	NKp46 High/MHC-I High vs. NKp46 Low/MHC-I High	0.1949
	NKp46 High/MHC-I Low vs. NKp46 Low/MHC-I High	0.1493
	NKp46 High/MHC-I Low vs. NKp46 Low/MHC-I Low	0.0949
	NKp46 Low/MHC-I High vs. NKp46 Low/MHC-I Low	0.5022

Supplemental Figures

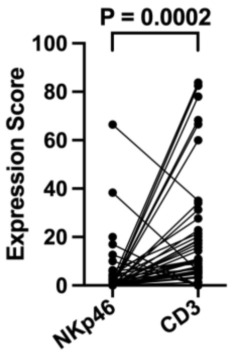

Supplemental Figure 1. Paired analysis of NKp46 and CD3 expression scores by IHC showing significantly greater levels of CD3 expression (P=0.0002) than NKp46, and an inverse relationship between the two immune subsets.

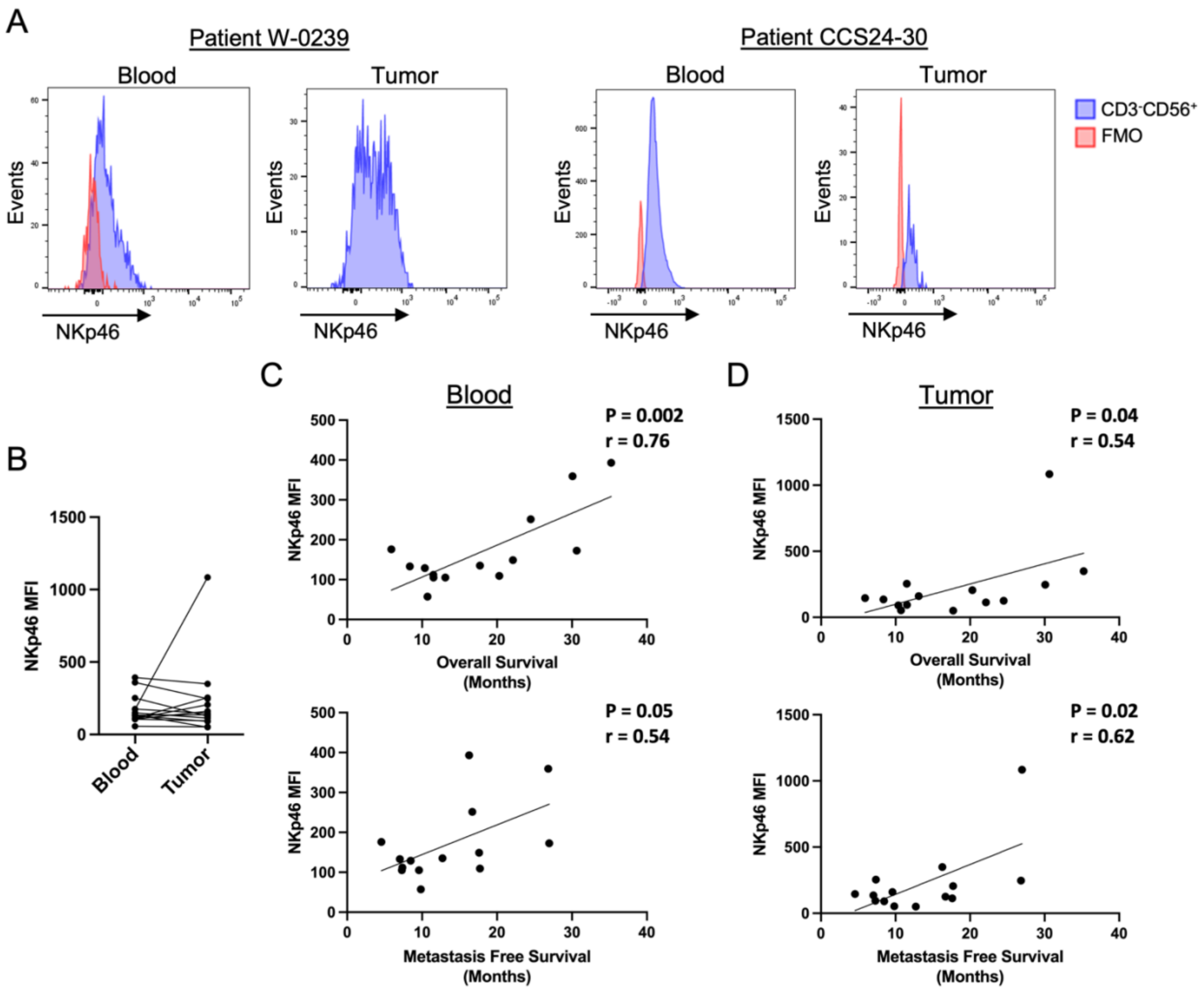

Supplemental Figure 2. NKp46 median fluorescence intensity (MFI) on NK cells is correlated with superior overall and metastasis free survival in soft tissue sarcoma. (A) Representative histograms from patients W-0239 and CCS24-30 of NKp46 MFI on $CD3^{-}CD56^{+}$ cells (blue) in blood and tumor compared with FMO control (red). (B) Paired analysis of NKp46 MFI showing no differences between blood and tumor (P=0.5). (C) NKp46 MFI demonstrating positive correlations with overall survival (P=0.002, r=0.76) and metastasis-free survival (P=0.05, r=054) in blood. (D) NKp46 MFI showing positive correlations with overall survival (P=0.04, r=0.54) and metastasis-free survival (P=0.02, r=062) in tumor. FMO, fluorescence minus one; MFI, median fluorescence intensity.

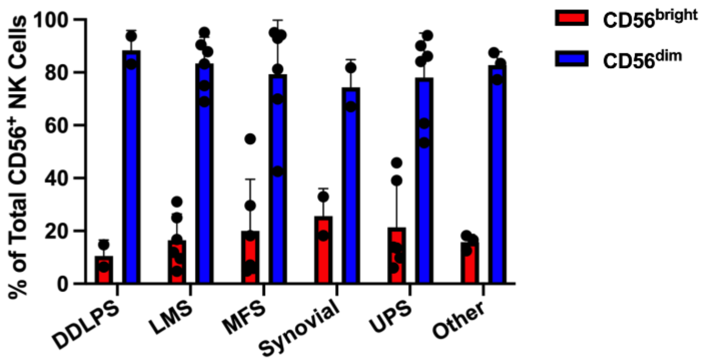

Supplemental Figure 3. CD56^{bright} and CD56^{dim} frequencies by histology subtypes showing higher CD56dim frequencies than CD56bright, regardless of histology subtype. DDPLS, dedifferentiated liposarcoma; LMS, leiomyosarcoma; UPS, undifferentiated pleomorphic sarcoma.

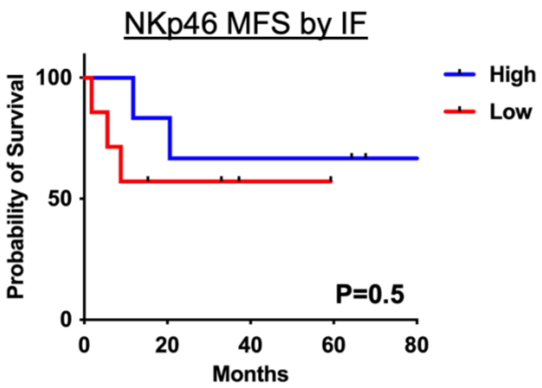

Supplemental Figure 4. Kaplan-Meier analysis of MFS stratified into high (N=6) versus low (N=8) NKp46 expression, stratified by the median, on IF showing superior MFS in patients with high NKp46 expression, though this did not reach statistical significance. IF, immunofluorescence; MFS, metastasis-free survival.

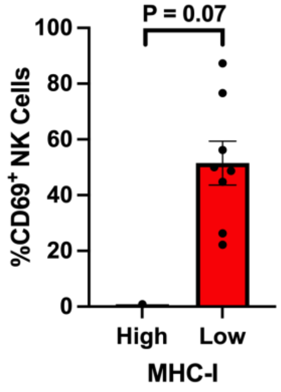

Supplemental Figure 5. Intratumoral %CD69^{+} NK cells by MHC-I expression levels showing greater %CD69^{+} NK cells in patients with low MHC-I expression, where high versus low was determined by the mean MHC-I expression on immunohistochemistry. Statistical analysis was performed with unpaired Student's t-test.
